# Supplementary material for: Impact of high body mass index on hepatocellular carcinoma risk in chronic liver disease: A population-based prospective cohort study
Source: PLoS One. 2025 Jan 22;20(1):e0316175. doi: 10.1371/journal.pone.0316175 (PMC11753674; doi:10.1371/journal.pone.0316175)
Supplement: S1 Table — (DOCX) [file pone.0316175.s001.docx]

S1 Table. Baseline demographic and clinical characteristics of patients with liver cirrhosis (n = 31,260)

| **Characteristic** | **HBV-LC**  **n = 6,403** | **HCV-LC**  **n = 863** | **ALD-LC**  **n = 3,073** | **NAFLD-LC**  **n = 654** | **Unspecified LC**  **n = 20,267** |
| --- | --- | --- | --- | --- | --- |
| HCC | 1,925 | 280 | 496 | 59 | 3,746 |
| Sex | | | | | |
| Men | 4,467 (69.8) | 460 (53.3) | 2,874 (93.5) | 470 (71.9) | 14,045 (69.3) |
| Women | 1,936 (30.2) | 403 (46.7) | 199 (6.5) | 184 (28.1) | 6,222 (30.7) |
| BMI, kg/m^2^ | | | | | |
| <18.5 | 125 (2.0) | 17 (2.0) | 140 (4.6) | 14 (2.1) | 637 (3.1) |
| 18.5–20.9 | 811 (12.7) | 94 (10.9) | 562 (18.3) | 56 (8.6) | 2,861 (14.1) |
| 21–22.9 | 1,462 (22.8) | 193 (22.4) | 750 (24.4) | 121 (18.5) | 4,607 (22.7) |
| 23–24.9 | 1,774 (27.7) | 268 (31.1) | 706 (23.0) | 168 (25.7) | 5,268 (26.0) |
| 25–27.4 | 1,507 (23.5) | 203 (23.5) | 596 (19.4) | 166 (25.4) | 4,515 (22.3) |
| 27.5–29.9 | 549 (8.6) | 63 (7.3) | 240 (7.8) | 94 (14.4) | 1,722 (8.5) |
| ≥30 | 175 (2.7) | 25 (2.9) | 79 (2.6) | 35 (5.4) | 657 (3.2) |
| Glycaemic status | | | | | |
| Normoglycemia | 4,297 (67.1) | 489 (56.7) | 1,374 (44.7) | 377 (57.6) | 11,912 (58.8) |
| IFG | 1,258 (19.6) | 177 (20.5) | 808 (26.3) | 140 (21.4) | 4,472 (22.1) |
| Diabetes | 848 (13.2) | 197 (22.8) | 891 (29.0) | 137 (20.9) | 3,883 (19.2) |
| Smoking, pack/day | | | | | |
| Never | 4,267 (66.6) | 641 (74.3) | 1,196 (38.9) | 400 (61.2) | 12,509 (61.7) |
| Former | 720 (11.2) | 74 (8.6) | 394 (12.8) | 71 (10.9) | 2,171 (10.7) |
| <0.5 | 445 (6.9) | 53 (6.1) | 471 (15.3) | 52 (8.0) | 1,744 (8.6) |
| 0.5–0.9 | 645 (10.1) | 50 (5.8) | 669 (21.8) | 93 (14.2) | 2,400 (11.8) |
| 1–1.9 | 171 (2.7) | 15 (1.7) | 275 (8.9) | 22 (3.4) | 911 (4.5) |
| ≥2 | 143 (2.2) | 28 (3.2) | 62 (2.0) | 14 (2.1) | 490 (2.4) |
| Unknown | 12 (0.2) | 2 (0.2) | 6 (0.2) | 2 (0.3) | 42 (0.2) |
| Alcohol, ethanol (g)/day | | | | | |
| None | 4,736 (74.0) | 685 (79.4) | 1,443 (47.0) | 380 (58.1) | 12,763 (63.0) |
| <10 | 960 (15.0) | 71 (8.2) | 429 (14.0) | 123 (18.8) | 3,180 (15.7) |
| 10–19 | 338 (5.3) | 35 (4.1) | 342 (11.1) | 74 (11.3) | 1,772 (8.7) |
| 20–39 | 97 (1.5) | 17 (2.0) | 221 (7.2) | 32 (4.9) | 711 (3.5) |
| ≥40 | 116 (1.8) | 13 (1.5) | 566 (18.4) | 29 (4.4) | 1,315 (6.5) |
| Unknown | 156 (2.4) | 42 (4.9) | 72 (2.3) | 16 (2.4) | 526 (2.6) |
| Physical activity, times/week | | | | | |
| None | 3,279 (51.2) | 477 (55.3) | 2,068 (67.3) | 356 (54.4) | 11,864 (58.5) |
| 1–2 | 1,809 (28.3) | 182 (21.1) | 525 (17.1) | 164 (25.1) | 4,457 (22.0) |
| ≥3 | 1,315 (20.5) | 204 (23.6) | 480 (15.6) | 134 (20.5) | 3,946 (19.5) |
| Income status, quartile | | | | | |
| Q1 (low) | 1,033 (16.1) | 170 (19.7) | 701 (22.8) | 106 (16.2) | 4,066 (20.1) |
| Q2 | 1,024 (16.0) | 146 (16.9) | 772 (25.1) | 110 (16.8) | 3,862 (19.1) |
| Q3 | 1,591 (24.8) | 200 (23.2) | 828 (26.9) | 174 (26.6) | 5,097 (25.1) |
| Q4 | 2,755 (43.0) | 347 (40.2) | 772 (25.1) | 264 (40.4) | 7,242 (35.7) |
| Age groups, years | | | | | |
| <65 | 5,944 (92.8) | 559 (64.8) | 2414 (78.6) | 538 (82.3) | 16,231 (80.1) |
| ≥65 | 459 (7.2) | 304 (35.2) | 659 (21.4) | 116 (17.7) | 4,036 (19.9) |
| Total cholesterol, mg/dL | | | | | |
| <200 | 4,987 (77.9) | 732 (84.8) | 2471 (80.4) | 379 (58.0) | 14,779 (72.9) |
| 200–239 | 1,152 (18.0) | 107 (12.4) | 446 (14.5) | 198 (30.3) | 4,072 (20.1) |
| ≥240 | 264 (4.1) | 24 (2.8) | 156 (5.1) | 77 (11.8) | 1,416 (7.0) |

Data are expressed as numbers and percentages.

Abbreviations: HBV, hepatitis B virus; LC, liver cirrhosis; HCV, hepatitis C virus; ALD, alcoholic liver disease; NAFLD, non-alcoholic fatty liver disease; HCC, hepatocellular carcinoma; BMI, body mass index; IFG, impaired fasting glucose
