# Supplementary material for: Impact of high body mass index on hepatocellular carcinoma risk in chronic liver disease: A population-based prospective cohort study
Source: PLoS One. 2025 Jan 22;20(1):e0316175. doi: 10.1371/journal.pone.0316175 (PMC11753674; doi:10.1371/journal.pone.0316175)
Supplement: S2 Table — (DOCX) [file pone.0316175.s002.docx]

S2 Table. Categorical analysis of the association between body mass index and the risk of hepatocellular carcinoma according to liver disease

| **Subgroup** | **Parameter** | **HR (95% CI)** | ***P*** |
| --- | --- | --- | --- |
| HBV | <18.5 | 1.02 (0.87–1.19) | 0.823 |
|  | 18.5–20.9 | 0.99 (0.92–1.06) | 0.763 |
|  | 21–22.9 | 1.04 (0.98–1.10) | 0.231 |
|  | 23–24.9 | 1.00 (reference) |  |
|  | 25–27.4 | 0.99 (0.94–1.05) | 0.747 |
|  | 27.5–29.9 | 1.09 (1.01–1.18) | 0.028 |
|  | ≥30 | 1.20 (1.06–1.36) | 0.004 |
| HCV | <18.5 | 0.69 (0.49–0.98) | 0.036 |
|  | 18.5–20.9 | 0.83 (0.70–0.99) | 0.037 |
|  | 21–22.9 | 0.90 (0.78–1.03) | 0.121 |
|  | 23–24.9 | 1.00 (reference) |  |
|  | 25–27.4 | 0.99 (0.86–1.13) | 0.837 |
|  | 27.5–29.9 | 1.09 (0.91–1.31) | 0.365 |
|  | ≥30 | 1.00 (0.73–1.36) | 0.975 |
| ALD | <18.5 | 1.14 (0.91–1.43) | 0.246 |
|  | 18.5–20.9 | 1.19 (1.05–1.35) | 0.007 |
|  | 21–22.9 | 1.28 (1.14–1.43) | <0.001 |
|  | 23–24.9 | 1.00 (Reference) |  |
|  | 25–27.4 | 1.00 (0.89–1.12) | 0.988 |
|  | 27.5–29.9 | 1.22 (1.06–1.42) | 0.007 |
|  | ≥30 | 1.38 (1.10–1.74) | 0.006 |
| NAFLD | <18.5 | 1.38 (0.72–2.61) | 0.329 |
|  | 18.5–20.9 | 1.11 (0.81–1.53) | 0.516 |
|  | 21–22.9 | 1.05 (0.82–1.34) | 0.694 |
|  | 23–24.9 | 1.00 (Reference) |  |
|  | 25–27.4 | 0.99 (0.80–1.22) | 0.921 |
|  | 27.5–29.9 | 1.36 (1.06–1.74) | 0.014 |
|  | ≥30 | 1.42 (1.00–2.01) | 0.051 |
| LC | <18.5 | 0.69 (0.57–0.82) | <0.001 |
|  | 18.5–20.9 | 0.83 (0.76–0.90) | <0.001 |
|  | 21–22.9 | 0.94 (0.88–1.01) | 0.078 |
|  | 23–24.9 | 1.00 (Reference) |  |
|  | 25–27.4 | 1.00 (0.93–1.07) | 0.992 |
|  | 27.5–29.9 | 1.11 (1.02–1.22) | 0.020 |
|  | ≥30 | 1.07 (0.93–1.24) | 0.362 |

Abbreviations: HR, hazard ratio; CI, confidence interval; HBV, hepatitis B virus; HCV, hepatitis C virus; ALD, alcoholic liver disease; NAFLD, non-alcoholic fatty liver disease; LC, liver cirrhosis
