# Supplementary material for: Impact of high body mass index on hepatocellular carcinoma risk in chronic liver disease: A population-based prospective cohort study
Source: PLoS One. 2025 Jan 22;20(1):e0316175. doi: 10.1371/journal.pone.0316175 (PMC11753674; doi:10.1371/journal.pone.0316175)
Supplement: S3 Table — (DOCX) [file pone.0316175.s003.docx]

S3 Table. Linear analysis of the association between body mass index and the risk of hepatocellular carcinoma according to the etiology of liver disease for each 5-kg/m^2^ BMI increase (BMI ≥25 kg/m^2^)

| **Subgroup** | **LC** | **Participants/HCC, n** | **HR (95% CI)** | ***P*** |
| --- | --- | --- | --- | --- |
| HBV | with LC | 2,231/739 | 1.19 (0.98–1.45) | 0.075 |
|  | without LC | 37,024/2,539 | 1.15 (1.03–1.27) | 0.011 |
| HCV | with LC | 291/90 | 1.38 (0.75–2.51) | 0.297 |
|  | without LC | 4,979/493 | 1.09 (0.86–1.37) | 0.486 |
| ALD | with LC | 915/162 | 1.36 (0.92–2.02) | 0.125 |
|  | without LC | 41,959/739 | 1.32 (1.10–1.58) | 0.003 |
| NAFLD | with LC | 295/25 | 1.59 (0.53–4.76) | 0.410 |
|  | without LC | 42,581/296 | 1.52 (1.17–1.97) | 0.002 |

Abbreviations: BMI, body mass index; LC, liver cirrhosis; HCC, hepatocellular carcinoma; HR, hazard ratio; CI, confidence interval; HBV, hepatitis B virus; HCV, hepatitis C virus; ALD, alcoholic liver disease; NAFLD, non-alcoholic fatty liver disease
