# Supplementary material for: Impact of high body mass index on hepatocellular carcinoma risk in chronic liver disease: A population-based prospective cohort study
Source: PLoS One. 2025 Jan 22;20(1):e0316175. doi: 10.1371/journal.pone.0316175 (PMC11753674; doi:10.1371/journal.pone.0316175)
Supplement: S4 Table — (DOCX) [file pone.0316175.s004.docx]

S4 Table. Cox regression analysis of hepatocellular carcinoma risk according to liver cirrhosis etiology, for each 5-kg/m^2^ BMI increase (BMI ≥25 kg/m^2^)

| **Subgroup** | **Participants/HCC, n** | **Parameter** | **HR (95% CI)** | ***P*** | ***P* for interaction** |
| --- | --- | --- | --- | --- | --- |
| HBV-LC | 2,231/739 | overall | 1.19 (0.98–1.45) | 0.075 |  |
|  |  | male | 0.95 (0.74–1.22) | 0.698 | 0.003^a^ |
|  |  | female | 1.73 (1.28–2.32) | <0.001 |  |
|  |  | <65 years | 1.17 (0.95–1.43) | 0.131 | 0.734^b^ |
|  |  | ≥65 years | 1.30 (0.72–2.35) | 0.378 |  |
| HCV-LC | 291/90 | overall | 1.38 (0.75–2.51) | 0.297 |  |
|  |  | male | 0.76 (0.26–2.19) | 0.612 | 0.127^a^ |
|  |  | female | 2.16 (0.95–4.95) | 0.067 |  |
|  |  | <65 years | 1.41 (0.66–3.01) | 0.381 | 0.247^b^ |
|  |  | ≥65 years | 0.60 (0.18–2.04) | 0.414 |  |
| ALD-LC | 915/162 | overall | 1.36 (0.92–2.02) | 0.125 |  |
|  |  | male | 1.43 (0.95–2.15) | 0.087 | 0.957^a^ |
|  |  | female | 1.35 (0.20–9.20) | 0.758 |  |
|  |  | <65 years | 1.31 (0.84–2.05) | 0.234 | 0.822^b^ |
|  |  | ≥65 years | 1.48 (0.57–3.87) | 0.424 |  |
| NAFLD-LC | 295/25 | overall | 1.59 (0.53–4.76) | 0.410 |  |
|  |  | male | 0.82 (0.13–5.18) | 0.831 | 0.408^a^ |
|  |  | female | 2.11 (0.59–7.58) | 0.253 |  |
|  |  | <65 years | 0.76 (0.19–3.05) | 0.699 | 0.036^b^ |
|  |  | ≥65 years | 11.08 (1.38–89.24) | 0.024 |  |

Abbreviations: BMI, body mass index; HCC, hepatocellular carcinoma; HR, hazard ratio; CI, confidence interval; HBV, hepatitis B virus; LC, liver cirrhosis; HCV, hepatitis C virus; ALD, alcoholic liver disease; NAFLD, non-alcoholic fatty liver disease

^a^ *P* for interaction between sexes

^b^ *P* for interaction between age groups
