# Supplementary material for: Impact of high body mass index on hepatocellular carcinoma risk in chronic liver disease: A population-based prospective cohort study
Source: PLoS One. 2025 Jan 22;20(1):e0316175. doi: 10.1371/journal.pone.0316175 (PMC11753674; doi:10.1371/journal.pone.0316175)
Supplement: Attachment — Submitted filename: review-comment-PONE-1130714.docx [file pone.0316175.s005.docx]

This is a large-scale, population-based study primarily exploring the impact of BMI on hepatocellular carcinoma (HCC) risk in patients with chronic liver diseases. While several studies have already shown that obesity and BMI can increase the risk of HCC, this study investigates various chronic liver diseases such as HBV, HCV, ALD, NAFLD (non-alcoholic fatty liver disease), and LC, as well as the effects of age and gender, providing new evidence. However, several points need further discussion.

Major comments:

1. What are the diagnostic criteria for alcoholic liver disease and non-alcoholic fatty liver disease? Are they based on case diagnosis codes?
2. Regarding Figure 3, is there statistical significance when the confidence interval crosses 1? For example, for patients under 65 years old, the odds ratio for NAFLD is 1.2 with a confidence interval of 0.87 to 1.64. Please confirm with a statistician.
3. In the discussion section for the BMI-HCC association stratified by age and gender, it is very complex and may not be easily understood by readers. Please make substantial revisions to this discussion section.

Minor comments:

1. On page 13, item 186, it should be Figure 2. Is this a typos?
2. On page 14, item 192, the title of Figure 3 is incorrect. Please revise it.
3. Please specify whether the definition of obesity based on BMI uses Western standards or Asian standards (BMI >23 or >25)
4. On page 18, item 287: the abbreviation full name of HLD ?
