# Supplementary material for: Impact of high body mass index on hepatocellular carcinoma risk in chronic liver disease: A population-based prospective cohort study
Source: PLoS One. 2025 Jan 22;20(1):e0316175. doi: 10.1371/journal.pone.0316175 (PMC11753674; doi:10.1371/journal.pone.0316175)
Supplement: Attachment — Submitted filename: Response to Reviewers.docx [file pone.0316175.s006.docx]

Journal Requirements:

1. When submitting your revision, we need you to address these additional requirements. Please ensure that your manuscript meets PLOS ONE's style requirements, including those for file naming. The PLOS ONE style templates can be found at

<https://journals.plos.org/plosone/s/file?id=wjVg/PLOSOne_formatting_sample_main_body.pdf> and

<https://journals.plos.org/plosone/s/file?id=ba62/PLOSOne_formatting_sample_title_authors_affiliations.pdf>

**Answer:** **We have carefully ensured that our manuscript adheres to PLOS ONE’s style requirements.**

2. We note that you have indicated that there are restrictions to data sharing for this study. For studies involving human research participant data or other sensitive data, we encourage authors to share de-identified or anonymized data. However, when data cannot be publicly shared for ethical reasons, we allow authors to make their data sets available upon request. For information on unacceptable data access restrictions, please see <http://journals.plos.org/plosone/s/data-availability#loc-unacceptable-data-access-restrictions>.

**Answer:** **We appreciate your understanding regarding the data restrictions. As previously noted, the data used in our study are owned by the National Health Insurance Service (NHIS) in Korea. As authors, we were granted access to the data at the NHIS office and were only permitted to take away the processed results. However, we are unable to share the raw data without permission from the NHIS. Regrettably, we are not authorized to share the raw data directly.**

3. When completing the data availability statement of the submission form, you indicated that you will make your data available on acceptance. We strongly recommend all authors decide on a data sharing plan before acceptance, as the process can be lengthy and hold up publication timelines. Please note that, though access restrictions are acceptable now, your entire data will need to be made freely accessible if your manuscript is accepted for publication. This policy applies to all data except where public deposition would breach compliance with the protocol approved by your research ethics board. If you are unable to adhere to our open data policy, please kindly revise your statement to explain your reasoning and we will seek the editor's input on an exemption. Please be assured that, once you have provided your new statement, the assessment of your exemption will not hold up the peer review process.

**Answer:** **As mentioned above, we would like to kindly present the following statement for the consideration of the editor and reviewers.**

“**Data availability: The data that support the findings of this study are available from the National Health Insurance Service (http://nhiss.nhis.or.kr/bd/ab/bdaba000eng.do), however, restrictions apply to the availability of these data, which were used under license for the current study, and so are not publicly available.”**

Reviewer #1: This is a large-scale, population-based study primarily exploring the impact of BMI on hepatocellular carcinoma (HCC) risk in patients with chronic liver diseases. While several studies have already shown that obesity and BMI can increase the risk of HCC, this study investigates various chronic liver diseases such as HBV, HCV, ALD, NAFLD (non-alcoholic fatty liver disease), and LC, as well as the effects of age and gender, providing new evidence. However, several points need further discussion.

**Answer: Thank you for your thoughtful summary of our study. We would like to express our sincere appreciation for carefully reviewing our paper and providing valuable feedback. Please find our point-by-point responses to your specific comments below.**

Major comments:

1. What are the diagnostic criteria for alcoholic liver disease and non-alcoholic fatty liver disease? Are they based on case diagnosis codes?

**Answer: Alcoholic liver disease (ALD) and non-alcoholic fatty liver disease (NAFLD) were defined using ICD-10 codes. The definition of ALD based on ICD-10 codes has been shown to have a strong dose-response relationship with alcohol consumption (see Yi SW et al., 2018, referenced below). Therefore, we believe this approach is reasonably valid for our study.
 Additionally, the diagnostic guidelines for ALD and NAFLD in South Korea are as follows:
 For alcoholic liver disease (ALD), diagnosis in South Korea is based on a daily alcohol intake of 40 g or more for men and 20 g or more for women, along with clinical, hematological, imaging, or histological evidence of liver disease, provided that other causes of liver damage are excluded. South Korea has established guidelines to diagnose ALD based on these criteria (2013 The Korean Association for the Study of the Liver, Alcoholic Liver Disease Guideline).
 Non-alcoholic fatty liver disease (NAFLD) is defined as liver disease in the absence of secondary causes such as significant alcohol consumption, drug-induced liver injury, or viral hepatitis, and is diagnosed based on clinical, biochemical, imaging, or pathological findings (2021 The Korean Association for the Study of the Liver, Alcoholic Liver Disease Guideline).**

- Yi SW, et al. Risk factors for hepatocellular carcinoma by age, sex, and liver disorder status: a prospective cohort study in Korea. Cancer. 2018;124: 2748–2757.

**According to the validation analysis of alcohol use performed in Yi SW et al.’s study (2018), the risk of ALD development directly increased with alcohol consumption, even in subgroups in which alcohol use was not associated with HCC risk, such as women, the youngest group (<50 years), the liver disorder group, and cirrhotic or HBV-infected people. For example, in women, the HRs for ALD were 3.7 (<10 g/day), 9.9 (10-39 g/day), and 31.3 (≥40 g/day) compared with non-users.)**

2. Regarding Figure 3, is there statistical significance when the confidence interval crosses 1? For example, for patients under 65 years old, the odds ratio for NAFLD is 1.2 with a confidence interval of 0.87 to 1.64. Please confirm with a statistician.

**Answer: We have revised Figure 3 to include the p-values as you suggested. We hope that this change better addresses your suggestion..**

3. In the discussion section for the BMI-HCC association stratified by age and gender, it is very complex and may not be easily understood by readers. Please make substantial revisions to this discussion section.

**Answer: We understand the importance of clarifying the age- and sex-related aspects of our discussion. As a result, we have made substantial revisions to present this section in a simpler and more concise manner.**

**“****Furthermore, our study showed a higher relative risk of HCC associated with BMI in women than men with viral hepatitis-LC co-occurrence. Few studies have examined sex-specific BMI-HCC associations in patients with viral hepatitis-LC co-occurrence [6,11, 25, 36-39]. In the current study, the BMI-HCC associations were similar between men and women with ALD; nevertheless, potentially stronger associations were observed in men than in women with NAFLD. Our study suggests different associations according to sex for each CLD. Unfortunately, except for the presumption of sex-specific differences in the hepatocarcinogenic mechanisms of high BMIs and each liver disease, proposing a mechanism that can adequately explain the sex differences in the obesity-related HCC risk observed in our study is difficult. Further studies are needed to clarify the potentially different associations according to sex for each CLD.**

**In the age-specific analysis, relative HCC risks associated with a higher BMI were higher in younger (<65 years) than in older adults with HBV and HCV infections, whereas the risks were higher in older than in younger adults with NAFLD. Younger patients with viral hepatitis may experience a stronger synergistic effect between viral infection and obesity due to lower comorbidity rates [14,40]. Regarding NAFLD, our finding of stronger associations in older adults may reflect the fact that high BMI affects HCC development in the long run. For HCC prevention, comorbid obesity may not be ignored in younger patients with viral hepatitis; however, more attention may be needed in NAFLD patients with obesity, especially in patients aged ≥65 years.”**

Minor comments:

1. On page 13, item 186, it should be Figure 2. Is this a typos?

**Answer:** **Apologies for the typo in our previous manuscript. It should have referred to Figure 2. We have now revised Figure 2 accordingly.**

2. On page 14, item 192, the title of Figure 3 is incorrect. Please revise it.

**Answer:** **Apologies for the typo in the titles. We have now revised.**

**Fig 2.** Hepatocellular carcinoma risk according to liver disease, for each 5-kg/m^2^ BMI increase in patients with BMI ≥25 kg/m^2^. HBV, hepatitis B virus; HCV, hepatitis C virus; ALD, alcoholic liver disease; NAFLD, nonalcoholic fatty liver disease; LC, liver cirrhosis; BMI, body mass index

**Fig 3.** Cox regression analysis of hepatocellular carcinoma risk according to liver disease, for each 5-kg/m^2^ BMI increase in patients with BMI ≥25 kg/m^2^. HBV, hepatitis B virus; HCV, hepatitis C virus; ALD, alcoholic liver disease; NAFLD, nonalcoholic fatty liver disease; LC, liver cirrhosis; HR, hazard ratio; CI, confidence interval

3. Please specify whether the definition of obesity based on BMI uses Western standards or Asian standards (BMI >23 or >25)

**Answer:** **We adhered to the World Health Organization's definitions for BMI (Obesity: BMI 30 or above; Overweight: BMI 25-29.9). Additionally, we have clarified the definitions of obesity and overweight as used in our study in the first paragraph of the Discussion section.**

4. On page 18, item 287: the abbreviation full name of HLD?

**Answer: It refers to “high-risk liver disease”, with the abbreviation first introduced in the Introduction section (Line 62, Page 5).**

Reviewer #2: In this study, Yi et al. investigated the association between BMI and HCC risk in patients with chronic liver disease. They found that higher BMIs were associated with increased HCC risks in patients with different etiologic chronic liver disease. Although the results showed clinically important, several points need be critically addressed.

**Answer: Thank you for your thoughtful and thorough summary of our study. We sincerely appreciate your careful review and the valuable feedback you have provided. Below, we have addressed each of your specific comments in a point-by-point manner.**

Specific comments
1. Current evidence showed that several viral factors before antiviral therapies are associated with the risk of HBV and HCV related HCC. In addition, antiviral therapies decrease the risk of HBV and HCV related HCC. The major limitations of this study include the lack of baseline HBV and HCV related viral factors and the data of antiviral therapy.

**Answer: Thank you for your valuable feedback. We acknowledge that antiviral therapies for HBV and HCV are significant factors in this context. Unfortunately, due to the limitations of our study, the baseline HBV and HCV related viral factors and the data of antiviral therapies were unavailable. We appreciate your understanding and the opportunity to address this limitation. Additionally, we have addressed this limitation in the discussion section of our manuscript as below. Thank you for pointing it out.**

**“Second, important data such as fibrosis score and antiviral therapies and detailed viral factors for HBV and HCV were not available. For example, antiviral therapies are known to reduce the risk of HBV- and HCV-related HCC.“**

2. The authors should describe the time point of baseline data collection.

**Answer:** **We appreciate your suggestion to clarify the time point. In response, we have revised the "Study population and follow-up" section to include the following explanation:**

**“Baseline data were collected at the time of the health examination (index date).”**

**Thank you for helping us improve the clarity of our manuscript.**

3. Body weight may change during the follow-up period. Only baseline BMI was used for analysis, the impact of changes of BMI on HCC risk cannot be estimated.
4. As above mention, the authors should consider using BMI as a time-dependent covariate to analyze the impact of the dynamic change of BMI on HCC risk.

**Answer:** **In response to questions 3 and 4, we appreciate your insights. We utilized BMI data from the participants' initial health check-ups, which was described as a limitation of our study in the revised manuscript as follows**:

**“Our analysis based on a single measurement of BMI may underestimate the true association due to regression dilution bias.”**

5. Regarding to the exclusion criteria, only 5062 patients with two or more chronic liver disease were excluded. Currently, the prevalence of NAFLD is so high, but the proportion of HBV or HCV combined with fatty liver is relatively low in this study. Please explain why?

**Answer:** **We acknowledge that the actual prevalence of NAFLD may be higher than reported in our study. However, NAFLD in our study was defined based on ICD-10 codes, which represent clinically diagnosed cases. As a result, cases of asymptomatic or mild NAFLD may have been excluded from our analysis. Thank you for noting this limitation.**

6. The diagnosis of patients was by the ICD-10 codes. How to confirm the coding is correct? For example, whether different doctors have the same diagnostic criteria for ALD.
**Answer: South Korea used to be an HBV endemic area. Serological tests for viral hepatitis have been introduced early in Korea and are commonly performed in individuals with suspected liver disease.** **Acute and chronic hepatitis B are diagnosed by detecting the hepatitis B virus surface antigen (HBsAg). Acute and chronic hepatitis C are diagnosed through the detection of anti-HCV antibodies or HCV RNA. In our previous study (Yi SW et al., 2018), HBV and HCV infections, defined by the same method as current study, increased HCC risk by around 20-fold in the multivariable-adjusted analysis after excluding cirrhosis. Therefore, diagnoses based on ICD-10 codes for viral hepatitis may be considered to be serologically accurate.
 For alcoholic liver disease (ALD) and Non-alcoholic fatty liver disease (NAFLD), South Korea has established guidelines to diagnose ALD and NAFLD (KASL. 2013, referenced below). Meanwhile, the definition of ALD based on ICD-10 codes has been shown to have a strong dose-response relationship with alcohol consumption (see Yi SW et al., 2018, referenced below). Therefore, we believe this approach is reasonably valid for our study**

- Yi SW, et al. Risk factors for hepatocellular carcinoma by age, sex, and liver disorder status: a prospective cohort study in Korea. Cancer. 2018;124: 2748–2757.
- KASL Clinical Practice Guidelines: Management of Alcoholic Liver Disease. Clin Mol Hepatol. 2013 Sep; 19(3): 216–254.
- KASL clinical practice guidelines: Management of nonalcoholic fatty liver disease. Clin Mol Hepatol. 2013 Dec; 19(4): 325–348.
