# Supplementary material for: Impact of high body mass index on hepatocellular carcinoma risk in chronic liver disease: A population-based prospective cohort study
Source: PLoS One. 2025 Jan 22;20(1):e0316175. doi: 10.1371/journal.pone.0316175 (PMC11753674; doi:10.1371/journal.pone.0316175)
Supplement: Attachment — Submitted filename: Response to Reviewers.docx [file pone.0316175.s007.docx]

Reviewer #2: This revised manuscript is improved and all previous comments were responded on point-to-point basis. However, the authors cannot analyze the impact of the dynamic change of BMI on HCC risk. The authors should provide more evidence to support that a single BMI measurement can determine the correlation with HCC.

**Answer:** Thank you for your thoughtful feedback. Please find our point-by-point responses to your specific comments below.

**We acknowledge that our analysis did not account for variations in BMI over time. Our data did not reveal changes in BMI throughout the study period.**

**Within the authors' review, a few studies examined the association between BMI (or Weight) change and HCC (or liver cancer) including studies of Li et al., (2013), Samantic et al., (2006), Schlesinger et al., (2013) and Yang et al., (JNCI Cancer Spectr, 2022). A summary of the key findings from each study is provided at the end of this response.**

**However, the studies mentioned above present inconsistent results and pose challenges in interpretation. Additionally, there are inherent difficulties associated with recalling past weight. Thus, research on weight change is considered a particularly challenging endeavor.**

**Consequently, in the limitations section, we have elaborated on the inability to account for variations in BMI over time, as follows.**

“Our analysis based on a single measurement of BMI may underestimate the true association due to regression dilution bias.”

-> **“We could not analyze the impact of the change of BMI on HCC risk. Only a few studies examined the association between BMI (or weight change) and HCC in persons with liver diseases (Li et al, 2013). The results were inconsistent. Our analysis based on a single measurement of BMI may underestimate the true association of dynamic change in BMI.”**

Thank you.

(1) Li Y et al. J Epidemiol. 2013 Body mass index and weight change during adulthood are associated with increased mortality from liver cancer: the JACC Study.

: In this study, Weight gain after age 20 (weight change 5 kg or more) among women with history of liver disease (83 liver cancer [LC] deaths) was associated with increased mortality from liver cancer, whereas no such increase was noted in men (338 LC deaths). Furthermore, among women without liver disease (90 LC deaths), weight gain did not correlate with an increase in liver cancer mortality. Weight change since age 20 years was calculated by subtracting self-reported weight at age 20 years from self-reported weight at baseline.

(2) Yang W et al. JNCI Cancer Spectr. 2022. Body mass index trajectories, weight gain, and risks of liver and biliary tract cancers

: In this study, individuals aged 20 to 50 who experienced a weight gain of 5 to less than 10 kg did not show an increased incidence of liver cancer compared to those with stable weight changes (-5 to less than 5 kg). Conversely, individuals with a weight gain of 10 kg or more exhibited an increased incidence of liver cancer. Meanwhile, weight gain from age 50 years to the study enrollment (mean age 62.6 years) did not have statistically significant associations with liver cancer (170 cases). Weight at recruitment was measured, while weight at age 20 and age 50 was recalled.

(3) Samanic C et al. Relation of body mass index to cancer risk in 362,552 Swedish men. Cancer Causes Control. 2006.

: The risks for liver cancer (33 cases) tended to increase with further increases in BMI over time, although p-value for each weight gain categories and p-value for trends were >0.05. Changes in measured BMI were calculated among 107,815 subjects with a 6-year follow-up exam.

(4) Schlesinger S et al. Abdominal obesity, weight gain during adulthood and risk of liver and biliary tract cancer in a European cohort. Int J Cancer. 2013

: Weight gain during adulthood was positively associated with HCC (108 cases) when comparing extreme tertiles. Weight change per year was computed as the difference between measured weight at recruitment and recalled weight at age 20 divided by time between age at recalled weight and baseline.

(5) Christie A Befort. JNCI Cancer Spectr. 2022. Obesity and Risk of Liver and Biliary Tract Cancer: Does Timing and Trajectory Matter?

: The editorial mentioned above notes that studies examining BMI changes predominantly rely on retrospective recall of weight at specific life stages (e.g., the 20-year mark in the Yang 2022 study). This approach fails to capture the complexities of weight management and potential weight regain following dieting throughout an individual's lifespan. Furthermore, the inherent uncertainties associated with weight recall may introduce confounding variables.
